# Rare disease awareness and perspectives of physicians in China: a questionnaire-based study

**DOI:** 10.1186/s13023-021-01788-3

**Published:** 2021-04-13

**Authors:** Xuefeng Li, Xiangyu Zhang, Shu Zhang, Zijuan Lu, Jianyong Zhang, Jincheng Zhou, Bingzhe Li, Li Ou

**Affiliations:** 1 Shenzhen Luohu People’s Hospital, The Third Affiliated Hospital of Shenzhen University, Shenzhen, 518001 People’s Republic of China; 2 Key Laboratory of Regenerative Biology, Guangdong Provincial Key Laboratory of Stem Cell and Regenerative Medicine, South China Institute for Stem Cell Biology and Regenerative Medicine, Guangzhou Institutes of Biomedicine and Health, Chinese Academy of Sciences, Guangzhou, 510530 People’s Republic of China; 3 The Sixth Affiliated Hospital of Guangzhou Medical University, Qingyuan People’s Hospital, Qingyuan, People’s Republic of China; 4 State Key Laboratory of Respiratory Disease, Sino-French Hoffmann Institute, School of Basic Medical Sciences, Guangzhou Medical University, Guangzhou, 511436 People’s Republic of China; 5 School of Statistics, University of Minnesota, Minneapolis, MN 55455 USA; 6 Department of Oral Implantology, The Affiliated Stomatology Hospital of Kunming Medical University, Kunming, 650106 People’s Republic of China; 7 School of Humanities, Tongji University, Shanghai, 200092 People’s Republic of China; 8 Jinhaishiji, 333 Jichanglu, Panzhihua, 617000 Sichuan People’s Republic of China; 9 Center for Design and Analysis, Amgen Inc, Thousand Oaks, CA 91320 USA; 10 School of Electrical and Computer Engineering, Oklahoma State University, Stillwater, OK 74078 USA; 11 Department of Pediatrics, Gene Therapy Center, University of Minnesota, Minneapolis, MN 55455 USA

**Keywords:** Rare disease, Questionnaire, Physicians, Diagnosis

## Abstract

**Background:**

It is estimated that there are over 16.8 million rare disease patients in China, representing a significant challenge for the healthcare system and society. Rare disease patients often experience delayed diagnosis, misdiagnosis, or improper treatment, which may be due to the lack of rare disease awareness among physicians.

**Materials and methods:**

A total of 224 physicians from different hospitals in China participated in the questionnaire, and 9 rare disease experts were interviewed with open-ended questions.

**Results:**

Most physicians (83.5%) were from Tertiary hospitals, which have over 500 beds. Only 5.3% of physicians were moderately or well aware of rare diseases. Most physicians (80.1%) had suspected their patients to have rare diseases less than 3 times. There was a strong support for special legislations for rare diseases and orphan drugs. Further, multinomial logistic regression (MLR) was used to determine whether hospitals, gender, and career length has an impact on perspectives and awareness. It was shown that male physicians were more likely to think newborn screening is important (*p* < 0.05). The longer the career length is, the more likely physicians believe that their previous education has not provided sufficient information about rare diseases and that their hospital has paid enough attention to rare diseases. Physicians from Tertiary A hospitals were more likely to rate the affordability of orphan drugs high. In addition, nine experts believed that rare disease awareness is essential for early diagnosis and timely treatment. These experts also made recommendations on how to improve rare disease awareness through medical school education and continuing training.

**Conclusions:**

Our study highlighted the importance of improving rare disease awareness among physicians in China. Recommendations about how to improve rare disease awareness in medical school education and establish an online ‘information hub’ are made for considerations of policy-makers.

**Supplementary information:**

The online version contains supplementary material available at 10.1186/s13023-021-01788-3.

## Background

It is estimated that there at least 16.8 million rare disease (by definition of Orphanet) patients in China, representing a significant challenge for the healthcare system that should not be neglected. In addition to the disease burden, rare disease patients often face the lack of treatment options, financial burden, and psychological stress [1]. Moreover, rare disease patients often experience delayed diagnosis: 5–6 years and 3–10 different physicians before a diagnosis [2–4]. Previous studies showed that approximately 30% of patients and families attributed delayed diagnosis to the lack of rare disease awareness and knowledge among physicians, as well as the limited availability of genetic testing [2, 3, 5]. Disease heterogeneity, variable presentation, and the lack of genetic data of rare diseases also contribute to difficulty in diagnosing rare diseases in China. As shown in a previous study, 78.8% of rare disease patients believed that they did not receive proper care due to the lack of rare disease awareness and knowledge among physicians [6].

The Chinese healthcare system consists of public and private hospitals and insurance programs. Although 95% of the population has basic health insurance coverage, public health insurance usually only covers approximately 50% of the medical cost, even less for serious or chronic conditions [7]. There are no national policies for rare diseases, and there also lacks national policies regarding newborn screening [8]. Although newborn screening has undergone a rapid development, it is still a great challenge to increase coverage and expand disease panel across the whole country [9]. Partially due to efforts of many patient organizations, rare disease awareness in China increased significantly [10]. Further, China released its first official list of 121 rare diseases in 2018, representing an initial and essential step. As a result, in recent years, there are more policies or initiatives aiming to support orphan drug development and accelerate evaluation and registration of orphan drugs. In summary, while there lacks policies and laws at the national level in China, there has been increased attention to rare diseases. In this study, we investigated rare disease awareness and perspectives among physicians and experts in China. The results generated will provide a basis for relevant departments to issue relevant policies, and provide reference for medical research and development.

## Results

### Questionnarie of physicians

A total of 1,802 physicians were contacted, and 224 (12.4% response rate) physicians participated in this study. The demographic information of these physicians is summarized in Table 1. Notably, it is difficult to estimate potential bias because information about physicians who decline to participate in this study and the general population of Chinese physicians is not available. However, the participants may represent a group of Chinese physicians who is more likely to have better rare disease awareness because the social media accounts and websites used to enroll participants have regular coverage of rare diseases.

**Table 1:** Demographic information of participants of the questionnaire for physicians

Gender	Male (n = 104, 46.4%)
	Female (n = 120, 53.6%)
Age (years)	< 30 (n = 34, 15.2%)
	30–45 (n = 132, 58.9%)
	46–60 (n = 52, 23.2%)
	> 60 (n = 6, 2.7%)
Hospital	Tertiary A (n = 163, 72.8%)
	Tertiary B (n = 16, 7.1%)
	Tertiary C (n = 8, 3.6%)
	Secondary A (n = 22, 9.8%)
	Secondary B (n = 1, 0.4%)
	Primary A (n = 4, 1.8%)
	Primary B (n = 4, 1.8%)
Career length (years)	< 5 (n = 49, 22.1%)
	5–15 (n = 81, 36.5%)
	16–30 (n = 71, 32.0%)
	> 30 (n = 21, 9.5%)
Specialty	Pediatrics (n = 13, 6.3%)
	Radiology (n = 22, 10.6%)
	Surgery (n = 18, 8.7%)
	Orthopedics (n = 10, 4.8%)
	Internal Medicine (n = 10, 4.8%)
	Dermatology (n = 10, 4.8%)
	Cardiology (n = 9, 4.3%)
	Nephrology (n = 5, 2.4%)
	Neurology (n = 5, 2.4%)
	Obstetrics and Gynecology (n = 8, 3.9%)
	Oncology (n = 9, 3.9%)
	Other (n = 88, 42.5%)

Only specialties with more than 4 participants were shown. Hospitals in China are organized into a 3-tier system: Primary (< 100 beds), Secondary (100–500 beds), or Tertiary institutions (> 500 beds). Moreover, based on size, medical equipment, medical technology, management, service provision, and medical quality, each tier are further divided into 3 levels: A, B and C. The category of other in specialty includes those with less than 5 participants, e.g., Chinese medicine, toxicology, dental, respiratory, molecular diagnostics, rehabilitation, urgent care, pathology, reproduction, otorhinolaryngology, ophthalmology, psychiatry, geriatrics, occupational diseases, endocrinology, pharmacology, public health, and anesthesiology

Out of 224 physicians, 104 (46.4%) were male and 120 (53.6%) are female while 132 (58.9%) were between 30 and 45. Hospitals in China are organized according to a 3-tier system that recognizes a hospital's ability to provide medical care, medical education, and conduct medical research. Based on this, hospitals are designated as Primary (< 100 beds), Secondary (100–500 beds), or Tertiary institutions (> 500 beds). Further, based on the level of service provision, size, medical technology, medical equipment, and management and medical quality, these 3 grades are further subdivided into 3 subsidiary levels: A, B and C. Out of 224 physicians, 163 (72.8%) were from Tertiary A (the highest level). Out of 222 physicians responded, 81 (36.5%) practiced between 5 and 15 years, and 71 (32.0%) practiced between 16 and 30 years.

**Fig.1:**
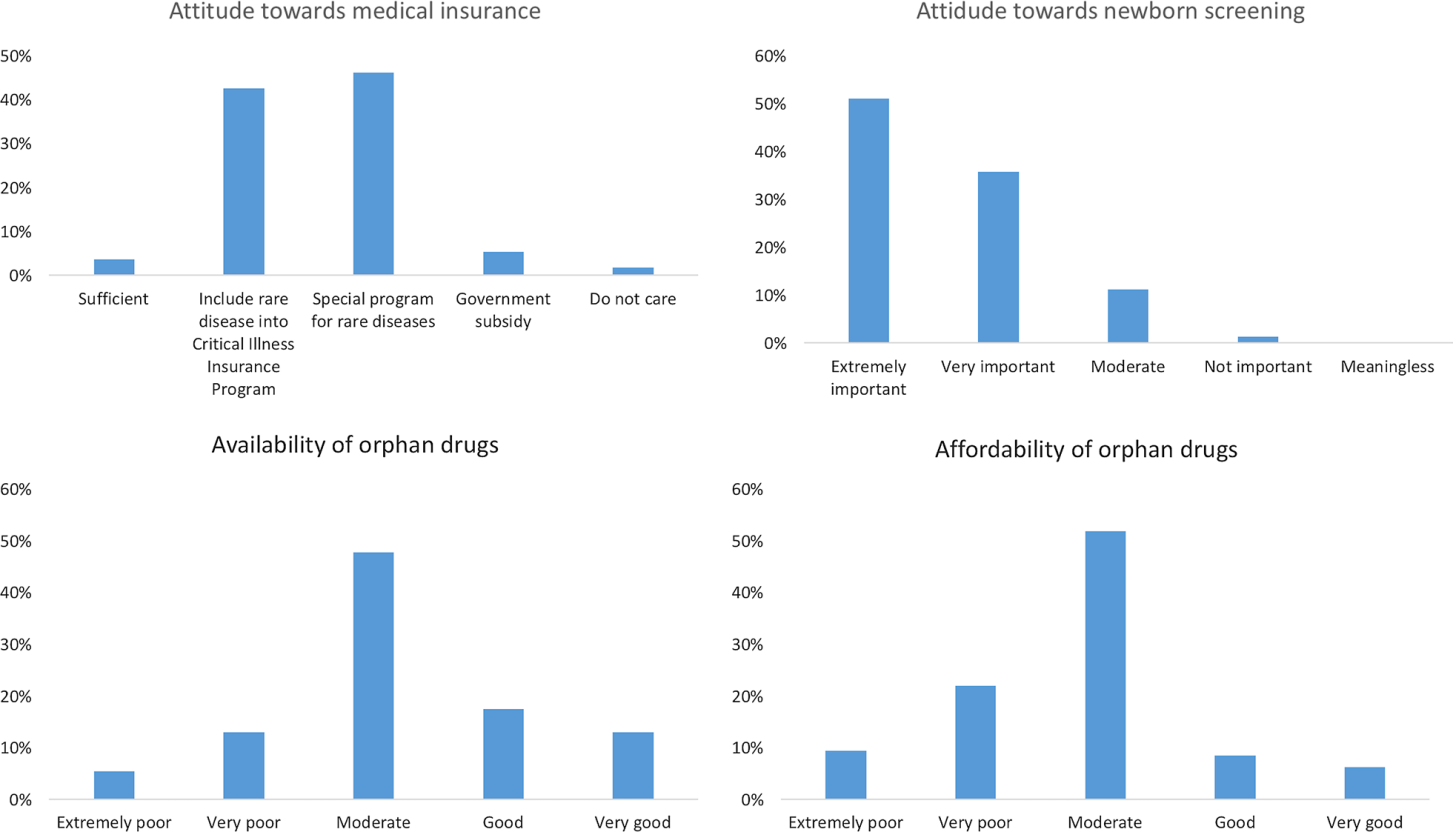
Attitude of physicians towards orphan drugs, medical insurance, and newborn screening

As to self-evaluated level of rare disease awareness, only 11 (4.9%) ‘moderately aware of’ rare diseases, and 1 (0.4%) ‘well aware of’ rare diseases. On the question ‘Have you ever suspected that a patient suffered from a rare disease?’, only 44 (19.9%) chose ‘over three times’. Out of 220 respondents, only 20 (9.1%) thought over 10% of their patients had rare diseases. Out of 218 respondents, 203 physicians (93.1%) thought rare disease patients were more difficult to manage. Out of 221 respondents, 133 thought their hospitals had not paid enough attention to rare disease patients. Out of 222 respondents, 119 (53.6%) physicians encountered one to five types of rare diseases. Out of 182 respondents, 89 physicians (48.9%) had no rare disease patients in the past year. Out of 177 respondents, 112 physicians (63.3%) had not treated any rare disease patients. Out of 222 respondents, 131 physicians (59%) ‘strongly’ supported special legislations for rare diseases, and 84 (37.8%) ‘moderately’ supported. These results highlighted the urgent need and wide support for special legislations for rare diseases. Out of 219 respondents, 126 physicians (57.5%) ‘strongly’ supported special legislations for orphan drugs, and 80 (36.5%) ‘moderately’ supported. Out of 222 respondents, only 8 (3.6%) thought that the Basic Medical Insurance Systems for Urban and Rural Residents is sufficient, and 4 (1.8%) suggested special subsidies from the government (Fig. 1a). Out of 222 respondents, only 3 (1.4%) rated it as ‘not that important’ (Fig. 1b). Out of 216 respondents, 107 physicians (49.5%) rated the availability of orphan drugs as ‘moderate’ (Fig. 1c). As to the affordability of orphan drugs, only 14 (6.4%) and 19 (8.7%) selected ‘very good’ and ‘good’, respectively. Out of 222 respondents, 195 (87.8%) selected ‘yes’ to the question ‘do you need information about rare diseases?’ As to the question ‘does your previous education and training provide sufficient information about rare diseases?’, out of 222 respondents, only 45 (20.3%) and 15 (6.8%) selected ‘very useful’ and ‘sufficient’, respectively.

**Fig.2:**
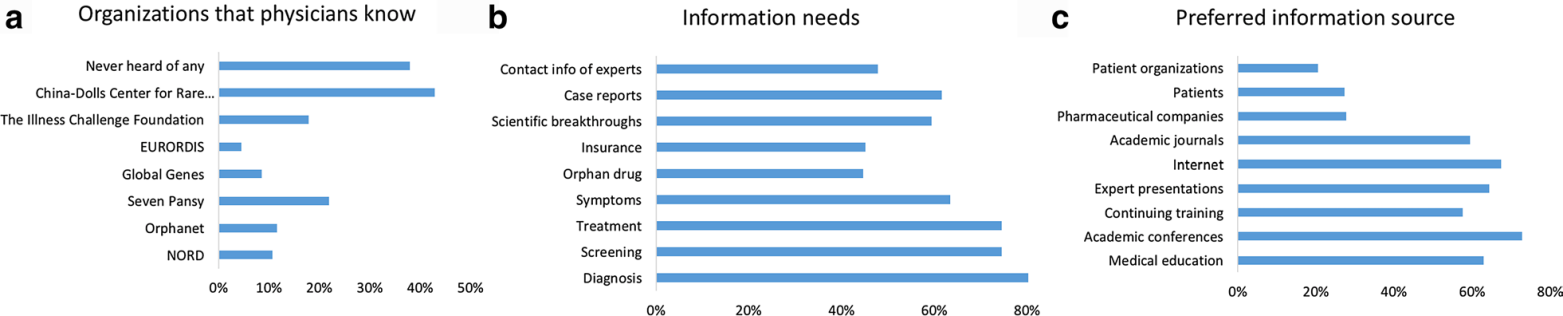
Information needs of physicians. **a** Patient organizations that physicians had heard of. **b** Information needs of physicians. **c** The preferred information source of physicians

As to organizations of rare disease patients, 96 physicians heard about China-Dolls Center for Rare Disorders, a non-government organization established in 2008 for patients with osteogenesis imperfecta. Forty-nine physicians heard about the Seven Pansy Rare Diseases Community, a national organization in China dedicated to raise rare disease awareness and provide scientific information and support. As shown in Fig. 2a, international sources, e.g., NORD (10.7%), Orphanet (11.6%), Global Genes (8.5%), EURORDIS (4.5%), were not as well-known in China. As to ‘What kind of information do you need?’, 186 respondents (83.0%) chose ‘diagnosis’, 167 (74.6%) for both ‘screening’ and ‘treatment’ (Fig. 2b). Interestingly, 106 out of 220 respondents (48.1%) only wanted to know information about rare diseases that can be possibly cured. As to the information source, 163 (72.8%) selected academic conferences, 152 (67.9%) chose the internet, 141 (62.9%) selected medical school education (Fig. 2c). Interestingly, only 61 (27.2%) selected patients, and 46 (20.5%) selected patient organizations.

Multinomial logistic regression (MLR) was used to determine whether hospitals, gender, and career length has an impact on perspectives and awareness (results summarized in Table 2). As to rare disease awareness, although age is better predictor with an Akaike information criterion (AIC) value of 456.604, no factors had a significant impact. This may be because few physicians were moderately or well aware of rare diseases, which would affect assumptions of MLR. As to Q11 (does your hospital pay enough attention to rare disease patients?), only career length has a significant impact with an AIC value of 289.4388, and *p* value of 0.0009. The longer their career length is, the more likely physicians have a positive response. As to Q12 (how many rare disease patients have you met?), gender has significant impact (AIC = 491.425, *p* = 0.02). Specifically, compared to ‘ > 10’, female physicians were more likely to respond ‘none’. As to Q13 (do you support special legislation for rare diseases?), no factors had a significant impact. Similarly, no factors had a significant impact on Q16 (do you support special legislations of orphan drugs?), Q17 (perspectives on medical insurance), Q18 (the availability of orphan drugs), and Q21 (do you need rare disease information?). Interestingly, hospitals had a significant impact on Q19 (affordability of orphan drugs) with an AIC value of 570.3079 and a *p* value of 0.017. Physicians from Tertiary A hospitals were more likely to rate the affordability of orphan drugs high. This may be explained by the fact that Tertiary A hospitals had more resources and insurance subsidies from the government. As to perspectives on newborn screening (Q20), gender and career length had significant impact (AIC = 442.2492, *p* < 0.05). Female physicians were less likely to think newborn screening is important. Notably, very few respondents believed newborn screening to be useless, which may affect assumptions of MLR. As to Q22 (does your previous education and training provide sufficient information about rare diseases?), career length was a good predictor (AIC = 612.4972, *p* < 0.05). The longer the career length is, the less likely they believe previous education to be useful. This may be explained by the fact that the amount of information about rare diseases in medical school education and job training had gradually increased over time. As to Q25 (do you only want information about rare diseases that can be possibly cured?), career length was also a good predictor (AIC = 290.00995, *p* < 0.01). Interestingly physicians with less than 5 years or over 30 years of experience were more likely to respond positively, while physicians with 5–30 years of experience had no preference.

**Table 2:** Impact of demographic information on rare disease awareness and perspectives

Rare disease awareness	No factors with statistical significance
Does your hospital pay enough attention to rare disease patients?	Career length (AIC = 289.4388, *p* = 0.0009
	The longer their career length was, the more likely physicians had a positive response
How many rare disease patients have you met?	Gender (AIC = 491.425, *p* = 0.02)
	Compared to ‘ > 10’, female physicians were more likely to respond ‘none’
Do you support special legislation for rare diseases?	No factors with statistical significance
Do you support special legislation of orphan drugs?	No factors with statistical significance
Perspectives on medical insurance	No factors with statistical significance
Availability of orphan drugs	No factors with statistical significance
Do you need rare disease information?	No factors with statistical significance
Affordability of orphan drugs	Hospital (AIC = 570.3079, *p* = 0.017)
	Physicians from Tertiary A hospitals were more likely to rate the affordability of orphan drugs high
Perspectives on newborn screening (AIC = 442.2492, *p* < 0.05)	Gender (AIC = 442.2492, *p* < 0.05)
	Female physicians were less likely to believe newborn screening to be important
Does your previous education and training provide sufficient information about rare diseases?	Career length (AIC = 612.4972, *p* < 0.05)
	The longer the career length was, the less likely they believe previous education to be useful
Do you only want information about rare diseases that can be possibly cured?	Career length (AIC = 290.00995, *p* < 0.01)
	Physicians with less than 5 years or over 30 years of experience were more likely to respond positively, while physicians with 5–30 years of experience had no preference

First, in Q4, hospitals were initially categorized into 9, and 72.8% of physicians were in the Tertiary A hospitals. Therefore, hospitals were re-categorized into Tertiary A and non-Tertiary A hospitals to ensure that each category has sufficient samples. Then, age and career length were correlated (R^2^ = 0.79). To avoid collinearity, only gender, hospital, and career length were analyzed as independent variables. Rare disease awareness and perspectives (listed in column 1) were analyzed as dependent variables. Then, a MLR analysis was performed with R. Akaike information criterion, AIC

### Interviews of rare disease experts

A total of 9 experts in the field of rare diseases were interviewed with exploratory questions. Notably, all of these experts were from Tertiary A hospitals located in big cities, such Beijing, Shanghai and Chengdu. As to how rare disease awareness facilitates fast and accurate diagnosis, most experts (7/9, 77.8%) agreed that such an awareness will point physicians to the right direction and then with additional information they may be able to reach a diagnosis. All experts agreed that physicians generally lacked the rare disease awareness, which was a major reason of misdiagnosis. Three experts (33.3%) suggested to establish a rare disease referral hub in each hospital, which collects potential rare disease patients and refer them to larger hospitals.

All experts believed that current medical school education had not provided enough information about rare diseases. Five experts (55.6%) recommended that in medical school education and continuing training, topics about a specific type of pathology and related common diseases should also mention potential related rare diseases. Also, case studies of the diagnosis pathway of a rare disease were suggested by seven experts. All experts believed that it would not be practical to include each rare disease in medical school education or continuing medical education. Therefore, the consensus is to adopt a method based on principles and organization of knowledge. Instead of solely teaching students the knowledge of rare diseases, more focus should be put on the ability to exclude common diseases and collect relevant information for diagnosis, as well as the knowledge of how to find information about treatment options upon diagnosis.

As to information sources of rare diseases, seven experts listed medical literature as the major source. Alternative sources included academic conferences, the internet, and patients. Interestingly, all experts mentioned the concept of ‘learning from the patients’, while only 20.7% of physicians listed patients as their information source in the questionnaire. These experts believed that rare disease patients were extremely active in learning about their conditions and scientific breakthroughs in the field, and thus sometimes could teach physicians much. One expert mentioned the experience of one patient explaining the relative strength and weakness of AAV gene therapy versus gene editing for Pompe disease. In general, these experts believed that current information source is not comprehensive and mainly targets experts instead of front line physicians. Six experts (66.7%) expressed that an ideal information source would be a search engine that one can submit symptoms and test results, and obtain possible diseases and treatment options in return. The output could also be recommendations on additional tests and evidence one should look for. Contact information of potential experts or hospitals for referral would be beneficial as well. Another feature of this search engine is a review system that other users can upvote, downvote, and comment on the output so that it can be optimized. Additionally, the search engine should be fully accessible to at least physicians with or without a cost.

All experts expressed the concern for the high cost of orphan drugs, especially gene therapy. Nevertheless, all of them acknowledged the cost involved for developing and manufacturing an orphan drug. All experts believed that legislation of an orphan drug act in China will remarkably accelerate orphan drug development and potentially lower the cost. Similar to the 224 physicians in the questionnaire, most experts (7/9, 77.8%) believed that a special rare disease insurance program would be the best option. One expert mentioned the Orphan Reinsurer and Benefit Manager (ORBM), a program being proposed in the United States to address the ultra-high cost of orphan drugs. Under the ORBM, payment is carved out by the primary insurer, which may be vulnerable to financial challenges of orphan drugs. ORBM aims to address the issue of payment timing, therapeutic performance risk, and actuarial risk [11].

Experts also commented on the biggest challenge when handling rare disease patients. One common issue is the lack of rare disease awareness among patients, families, and many frontline physicians. Symptoms of many rare diseases are non-obvious and even difficult to explain, which are thus often ignored, resulting in significant delay in diagnosis and management. Another difficulty is the lack of reliable, up-to-date, and accessible information source. Most experts (8/9, 88.9%) believed that the language barrier creates much challenge while physicians searching for relevant information. Although international organizations, such as Orphanet, NORD, established systems of reliable and comprehensive information about rare diseases, it is not readily accessible for Chinese physicians, most of whom do not speak English. Further, although only 19% of physicians the questionnaire rated the availability of orphan drugs as ‘poor’ or ‘very poor’, all experts expressed the concern over the lack of treatment options. As to the discrepancy, seven experts believed that it was because ‘most physicians do not know the existence of orphan drugs in Western countries’.

## Discussion

Only 12 (5.3%) ‘moderately or well aware of’ rare diseases. A previous study in Spain showed that 15% of physicians have a good knowledge about rare diseases [12], significantly better than a total of 5.3% in this study. These results indicated a significant lack of awareness of rare diseases among physicians in China. Considering the fact that most physicians were from Tertiary hospitals with > 500 beds and located in big cities, the rare disease awareness among average physicians in China would be even worse. The combined total of physicians who encountered rare disease patients at least once was 83.3%, similar to the number among physicians in Spain (90%) [13]. Out of 222 respondents, 195 (87.8%) selected believed that they needed information about rare diseases, similar to a previous study in Belgium (83–97%) [14]. International information sources, e.g., NORD, Orphanet, and Global Genes, were not well-known in China. It was shown that among pediatricians in Australia, the percentage of awareness of Orphanet, NORD, and EURORDIS was 50%, 35%, and 21%, respectively [15]. This discrepancy may be partly due to the language barrier.

In the interviews, some experts suggested to establish a rare disease referral hub in each hospital, which collects potential rare disease patients and refer them to larger hospitals. The majority of Chinese population do not live in big cities that have large hospitals and experts. Also, it is unrealistic to provide comprehensive rare disease information to all physicians in smaller hospitals. Such a rare disease referral hub is not expected to incur significant burden to hospitals, therefore, it is feasible and should significantly help the diagnosis and treatment of rare diseases. All experts believed that current medical school education had not provided enough information about rare diseases. The French National Plan for Rare Diseases stipulated that medical school curriculum should include education in rare diseases [16]. Such stipulations do not exist in China, but all experts recommended inclusion of rare disease topics in medical school curriculum. All experts expressed the concern for the high cost of orphan drugs, especially gene therapy. Nevertheless, all of them acknowledged the cost involved for developing and manufacturing an orphan drug. In 1983, the United States passed the Orphan Drug Act, which provide tax incentives, enhanced patent protection and marketing right, and clinical research subsidies to pharmaceutical companies that develop orphan drugs. The number of orphan drugs significantly from 38 before 1983 to over 600 in 2019. Many other countries and districts pass similar laws to stimulate orphan drug development. All experts believed that legislation of an orphan drug act in China will remarkably accelerate orphan drug development and potentially lower the cost.

At present, there are more than 7,000 rare diseases known in the world, of which only 5% are medicated; among the 350 million rare disease patients in the world, there are estimated to be 16.8 million rare disease patients in China [17]. However, due to insufficient disease awareness and lack of effective treatment options, patients with rare diseases have long faced the dilemma of low diagnosis and treatment rates. It is urgent to popularize disease knowledge and improve the level of diagnosis and treatment of rare diseases [18]. Due to the extremely low prevalence and incidence, the small number of patients, and the scattered population of patients, most rare diseases require multidisciplinary, interdisciplinary clinical experts and medical genetic experts only through collaboration can accurate diagnosis and treatment be achieved [19]. Thus, empowering physicians is a huge project that requires more support and help from government departments and all walks of life. Major hospitals should establish regular rare disease diagnosis expert teams and formulate scientific and effective training plans so that doctors will no longer have nothing to do with rare diseases.

The establishment of a rare disease registration platform can form a rare disease knowledge base, realize multi-level sharing of rare disease data, reduce the time for physicians to diagnose, and improve the accuracy of diagnosis [20]. At the same time, the establishment of the system is conducive to the formation of unified rare disease registration technical standards and norms, uniting superior units to form a collaborative network of rare diseases, carrying out rare disease registration research nationwide, and establishing a rare disease direct reporting system [21]. The establishment of a new genetic counseling system will also help patients with rare diseases find and diagnose them as soon as possible. Previous report has pointed out that about 80% of rare diseases are genetic, which means that most undiagnosed patients need to consult a medical geneticist to shorten the diagnosis time [21]. Through the establishment of pre-diagnosis centers in genetic clinics or information collection and remote consultation for patients in rural and remote areas, patients can quickly and effectively obtain genetic diagnosis and consultation. At the same time, genetic diagnosis and consultation also require multidisciplinary and interdisciplinary clinical experts. According to industry insiders, my country currently lacks medical talents with multidisciplinary development. Training genetic counselors can solve the problems caused by the shortage of talents in this area [22]. In addition, applying in silico tools for genotype–phenotype correlation has the potential to significantly help diagnosis and prognosis [23].

In China, due to the imbalance of medical information and resources, the many basic medical needs of patients with rare diseases urgently need to be met. It is even more necessary to rely on the help of patient organizations to provide a patient in seeking medical advice, knowledge popularization, patient care, and rehabilitation support. A platform for communication and voice, appealing to all sectors of society to pay attention to and support the group of patients with rare diseases.

## Conclusions

These results highlight the importance of improving rare disease awareness among physicians in China. Recommendations about how to improve rare disease awareness in medical school education and establish an online ‘information hub’ are made for considerations of policy-makers.

## Methods

### Study design and participant enrollment

In January 2020, our group in cooperation with Seven-Pansy Rare Disease Community, Guangzhou Medical University, and Shenzhen University, invited physicians for a questionnaire and rare disease experts for interviews. Physicians were invited through social media, websites, or newsletters. All questionnaires were completed between March to April 2020. Rare disease experts were invited for an interview through Seven-Pansy Rare Disease Community, a national rare disease organization in Shanghai. All interviews were conducted over the phone between April to May 2020. The interviews were audio recorded with the interviewees’ permission and transcribed verbatim for further analysis. The questionnaire and open-ended questions for interviews were included in the supplementary materials.

### Ethics, consent, and permission

The study was approved by the Institutional Ethics Committee of Guangzhou Medical University. The study was approved by the Institutional Ethics Committee of the Guangzhou Medical University, China. A questionnaire and a list of open-ended questions for interviews were designed in Chinese. Potential participants were invited to participate in this study, and only those who signed the informed consent participated in this study. All the participants acknowledged: (1) the sponsor of the study; (2) the objectives of the study; (3) the affiliation of the investigators; (4) that they can decline to answer any of the questions; (5) that the information collected will only be used for academic research; (6) that they can quit the study at any time; (7) that the results will be published in a scientific journal without seeking their approval of the manuscript; (8) that they will participate in this study anonymously; and (9) that they will not be paid for participating in this study.

### Data analysis

To identify parameters that have impact on rare disease awareness and perspectives, the correlation between Q1-6 and Q7-15 were analyzed. First, in Q4, hospitals were categorized into 9, and 72.8% of physicians were in the Tertiary A hospitals. Therefore, hospitals were re-categorized into Tertiary A and non-Tertiary A hospitals to ensure that each category has sufficient samples. Then, age (Q3) and career length (Q5) was correlated (R^2^ = 0.79). To avoid collinearity, only gender (Q2), hospital (Q4), and career length (Q5) were included for analysis. In summary, gender, hospital, and career length were defined as independent variables, while rare disease awareness and perspectives were analyzed as dependent variables. R was used to analyze data, *p* value < 0.05 (two-tailed tests) was used to denote statistical significance. Multinomial logistic regression was used to determine parameters that have an impact on awareness and perspectives.

## Supplementary information

**Additional file 1.** List of questions.

## Data Availability

The datasets used and/or analyzed during the current study are available from the corresponding author on reasonable request.

## Abbreviations

NORD: National Organization for Rare Disorders
EURORDIS: European Organization for Rare Diseases
CORD: Canadian Organization for Rare Diseases
