# Supplementary material for: Rare disease awareness and perspectives of physicians in China: a questionnaire-based study
Source: Orphanet J Rare Dis. 2021 Apr 13;16:171. doi: 10.1186/s13023-021-01788-3 (PMC8042908; doi:10.1186/s13023-021-01788-3)
Supplement: Supplementary file 1 — Additional file 1. List of questions. [file 13023_2021_1788_MOESM1_ESM.docx]

**Questionnaires for physicians**

1. 您的性别? (Gender)

2. 您的年龄? (Age)

<30

30-45

45-60

>60

3. 您来自的医院属于? (What hospital are you affiliated with?)

三级甲等 (Tertiary A)

三级乙等 (Tertiary B)

三级丙等 (Tertiary C)

二级甲等 (Secondary A)

二级乙等 (Secondary B)

二级丙等 (Secondary C)

一级甲等 (Primary A)

一级乙等 (Primary B)

一级丙等 (Primary C)

4. 您的行医年数？(Career length)

5. 您是哪个科室的医生？(Specialty)

6. 您的病人当中只有不到10%是罕见病患者？(Less than 10% of your patients had rare diseases?)

是 (Yes)

否 (No)

不清楚 (Not sure)

7. 应对罕见病患者是否比其他患者困难？(Rare disease patients are more difficult to manage?)

是 (Yes)

否 (No)

8. 您所在的医院是否对罕见病患者提供了足够的关注？(Does your hospital pay enough attention to rare disease patients?)

是 (Yes)

否 (No)

9. 您对罕见病的了解 (Please rate your awareness of rare diseases)

从未听说 (Never heard of)

听说过，但不太了解 (heard of)

有一些了解 (knew a little)

比较了解 (moderately aware of)

非常了解 (well aware of)

10. 您遇到过多少种罕见病? (How many types of rare diseases have you encountered?)

>10

6-9

1-5

0

11. 在过去的一年里，您接诊过多少罕见病患者？(In the past year, how many rare disease patients have you encountered?)

12. 在过去的一年里，您治疗过多少罕见病患者？(In the past year, how many rare disease patients have you treated?)

13. 您以前接受的教育和培训对于您应对罕见病是否有用？(Does your previous education and training provide sufficient information about rare diseases?)

非常有用 (Very useful)

足够有用 (Sufficient)

一般有用 (Moderately useful)

不够有用 (Not sufficient)

完全没用 (Not useful at all)

14. 您支持专门的罕见病政策出台吗？(Do you support special legislations for rare diseases?)

非常支持 (Strongly support)

比较支持 (Moderately support)

不关心 (Do not care)

不支持 (Do not support)

15. 你支持出台孤儿药法案吗？(Do you support an orphan drug act in China?)

非常支持 (Strongly support)

比较支持 (Moderately support)

不关心 (Do not care)

不支持 (Do not support)

16. 罕见病的医保问题您有什么看法？(Comments on insurance for rare diseases?)

现有的城乡居民医保制度已经足够了 (Basic Medical Insurance Systems for Urban and Rural Residents is sufficient)

建议将罕见病纳入大病医保 (Inclusion of rare diseases into Major Illness Medical Insurance)

增设专门的罕见病医保项目 (Special insurance programs for rare diseases)

政府额外补贴 (Special subsidies from the government)

不关心 (Do not care)

17. 罕见病药物的可获得程度评价 (Please rate the availability of orphan drugs)

非常好 (Very good)

很好 (Good)

一般 (Mediocre)

不好 (Bad)

非常差 (Very bad)

17. 罕见病药物的可负担程度评价 (Please rate the affordability of orphan drugs)

非常好 (Very good)

很好 (Good)

一般 (Mediocre)

不好 (Bad)

非常差 (Very bad)

18. 您是否需要罕见病的相关信息？(Do you need information about rare diseases?)

是 (Yes)

否 (No)

19. 以下罕见病信息获取渠道（相关机构，资讯来源，搜索引擎和期刊），您听说过哪些？(Have you heard of any of the following information sources?)

NORD

Orphanet

EURORDIS

Global Genes

七色堇 (Seven-Pansy Rare Diseases Community)

病痛挑战基金会 (The Illness Challenge Foundation)

瓷娃娃罕见病关爱中心 (China-Dolls Center for Rare Diseases)

Orphanet Journal of Rare Diseases

都没听过 (Never heard of any)

20. 您需要哪些罕见病相关信息呢？(What kind of information do you need?)

诊断 (Diagnosis)

预防和筛选 (Screening)

治疗方案 (Treatment)

症状 (Symptoms)

孤儿药 (Orphan drug)

医保信息 (Insurance)

相关研究进展 (Scientific breakthrough)

临床案例 (Case reports)

领域内专家联系方式 (Contact info of experts)

21. 您是否只想了解哪些有可能治愈的罕见病的信息？(Do you only want to know information about rare diseases that can be possibly cured?)

是 (Yes)

否 (No)

22. 您希望从哪些渠道获得罕见病的信息？ (What kind of information source do you have?)

医学院教育 (Medical education)

学术会议 (Academic conferences)

在职培训 (Continuing training)

专家讲座 (Expert presentation)

网络 (Internet)

学术期刊 (Academic journals)

药企宣传 (Pharmaceutical companies)

患者 (Patients)

患者组织 (Patient organizations)

23. 您对罕见病的新生儿筛选有什么看法？(Comment on newborn screening

至关重要 (Essential)

很重要 (Important)

一般 (Mediocre)

不是很重要 (Not important)

没有意义 (Meaningless)

**Interview questions for rare disease experts**

1. 您认为对罕见病的认知如何加快诊断和减少误诊可能性？(How rare disease awareness helps accelerate diagnosis and minimize the possibility of misdiagnosis?)

2. 您认为医学院教育应该如何改进来提高对罕见病的认知？(How can medical education be improved to increase rare disease awareness?)

3. 您现在主要使用什么来增加对罕见病的了解？(What is your information source to increase your knowledge of rare diseases?)

4. 您认为什么是罕见病的最佳信息来源？为什么？(What is the ideal information source of rare diseases? Why?)

5. 理想中的罕见病网络平台应该具有哪些特点？(What characteristics an ideal online platform of rare diseases should have?)

6. 对于罕见病药物的高昂费用（如天价基因疗法）有什么看法？(What do you think of the ultrahigh cost of orphan drugs, especially gene therapy?)

7. 在诊断罕见病过程中的最大挑战是？(What is the biggest challenge of diagnosing a rare disease?)

8. 在治疗罕见病过程中的最大挑战是？(What is the biggest challenge of treating a rare disease?)
